# Research progress of CTC, ctDNA, and EVs in cancer liquid biopsy

**DOI:** 10.3389/fonc.2024.1303335

**Published:** 2024-01-25

**Authors:** Xiaoling Wang, Lijuan Wang, Haihong Lin, Yifan Zhu, Defa Huang, Mi Lai, Xuxiang Xi, Junyun Huang, Wenjuan Zhang, Tianyu Zhong

**Affiliations:** ^1^ Laboratory Medicine, First Affiliated Hospital of Gannan Medical University, Ganzhou, China; ^2^ The First School of Clinical Medicine, Gannan Medical University, Ganzhou, China

**Keywords:** CTC, ctDNA, EVs, cancer, liquid biopsy, biomarker, precision

## Abstract

Circulating tumor cells (CTCs), circulating tumor DNA (ctDNA), and extracellular vehicles (EVs) have received significant attention in recent times as emerging biomarkers and subjects of transformational studies. The three main branches of liquid biopsy have evolved from the three primary tumor liquid biopsy detection targets—CTC, ctDNA, and EVs—each with distinct benefits. CTCs are derived from circulating cancer cells from the original tumor or metastases and may display global features of the tumor. ctDNA has been extensively analyzed and has been used to aid in the diagnosis, treatment, and prognosis of neoplastic diseases. EVs contain tumor-derived material such as DNA, RNA, proteins, lipids, sugar structures, and metabolites. The three provide different detection contents but have strong complementarity to a certain extent. Even though they have already been employed in several clinical trials, the clinical utility of three biomarkers is still being studied, with promising initial findings. This review thoroughly overviews established and emerging technologies for the isolation, characterization, and content detection of CTC, ctDNA, and EVs. Also discussed were the most recent developments in the study of potential liquid biopsy biomarkers for cancer diagnosis, therapeutic monitoring, and prognosis prediction. These included CTC, ctDNA, and EVs. Finally, the potential and challenges of employing liquid biopsy based on CTC, ctDNA, and EVs for precision medicine were evaluated.

## 1 Introduction

Cancer is a highly lethal ailment that poses a significant threat to human existence. Key components of cancer prevention, detection, and therapy encompass the assessment of the effectiveness of cancer treatment, the surveillance of patient’s post-treatment, and the provision of timely alerts regarding the risk of tumor metastasis and recurrence. There is no accurate biomarker for early cancer diagnosis due to the complexity of tumor incidence and tumor heterogeneity, Despite the development of multiple technologies aimed at the early detection of tumor biomarkers from diverse clinical samples and the significant research conducted in this field (1). Technological developments have led to alternate methods of tumor liquid biopsy, which have seen significant success and promise (2). The current understanding in the scientific community is that a tumor can release many components, such as tumor cells proteins, extracellular vehicles (EVs), and nucleic acids, into the peripheral circulation (3). Since the original identification of tumor cells in patient peripheral blood in the 1860s, significant advancements have been made in separating circulating tumor cells (CTCs) from various blood cell types (4). Furthermore, in 1996, the identification of microsatellite alterations in the circulating tumor DNA of individuals with tumors demonstrated a precise correlation with the microsatellite changes noticed in the primary tumor (5).In 2011, the concept of liquid biopsy based on CTC detection was proposed (4), and after that, the technology of using ctDNA to obtain tumor biological information was included in the liquid biopsy’s scope (5).In 2015, the Massachusetts Institute of Technology Review named liquid biopsy one of the top ten breakthrough technologies of the year. Afterward, liquid biopsy entered a rapid development stage, and detection technologies such as extracellular vehicles (EVs) (6) and tumor-educated platelets (7) were also included in the scope of liquid biopsy. A method of *in vitro* diagnosis called liquid biopsy uses body fluids as the test material to obtain tumor biological information relative to tissue biopsy. Liquid biopsy, which obtains a sample of bodily fluids such as saliva, blood, and cerebrospinal fluid, has gained broad attention due to its little invasion and has increased prospects for cancer detection and continuous monitoring (6).

Each of the three techniques in liquid biopsy has its own strengths. CTCs detection monitors the trend of changes in the type and number of CTCs by capturing CTCs present in the peripheral blood in order to monitor tumor dynamics in real time, assess treatment efficacy, and enable real-time individual therapy. ctDNA is a fragment of DNA that is necrotic, apoptotic, or normally secreted by tumor cells into the bloodstream, and carries information about cancer-related genetic mutations, and can be used for early detection of cancer when the it can be used for early detection of cancer because it can be detected when mutations occur at the molecular level of the tumor. ctDNA is capable of fusion and remapping in different types of cancers (8). ctDNA has a number of clinical applications, for example, sequential ctDNA assays can be used to efficiently monitor patients and detect tiny residual lesions, which can help in the early detection of disease progression and the adjustment of the Adjuvant therapeutic regimens for ovarian cancer treatment (9). Plasma preoperative ctDNA testing in ovarian cancer patients is expected to serve as a biomarker for tumor staging and prognosis prediction (10). Early changes in circulating tumor DNA (ctDNA) predict treatment response in patients with metastatic KRAS-mutant colorectal cancer (mCRC) (11). ctDNA and radiated tumor volume identify patients with resected early-stage non-small-cell lung cancers who are at risk of recurrence (12). EVs have been linked to driving malignant cell behavior, including stimulating tumor cell growth, suppressing immune responses, inducing angiogenesis, facilitating tumor cell migration, and establishing metastasis, making them particularly attractive as cancer biomarkers (13). EVs contain cargoes of miRNAs,mRNAs, and proteins. It has been shown that microRNAs carried by EVs are associated with colorectal cancer (14), hepatocellular carcinoma (15), lung cancer (16), oral cancer (17), and ovarian cancer (18). The contents of EVs such as mRNA can promote epithelial-mesenchymal transition and chemoresistance in colorectal cancer (19). Therefore, not only CTCs can be used for tumor liquid biopsy, but also ctDNA,EVs can be used for cancer detection.

**Figure 1:**
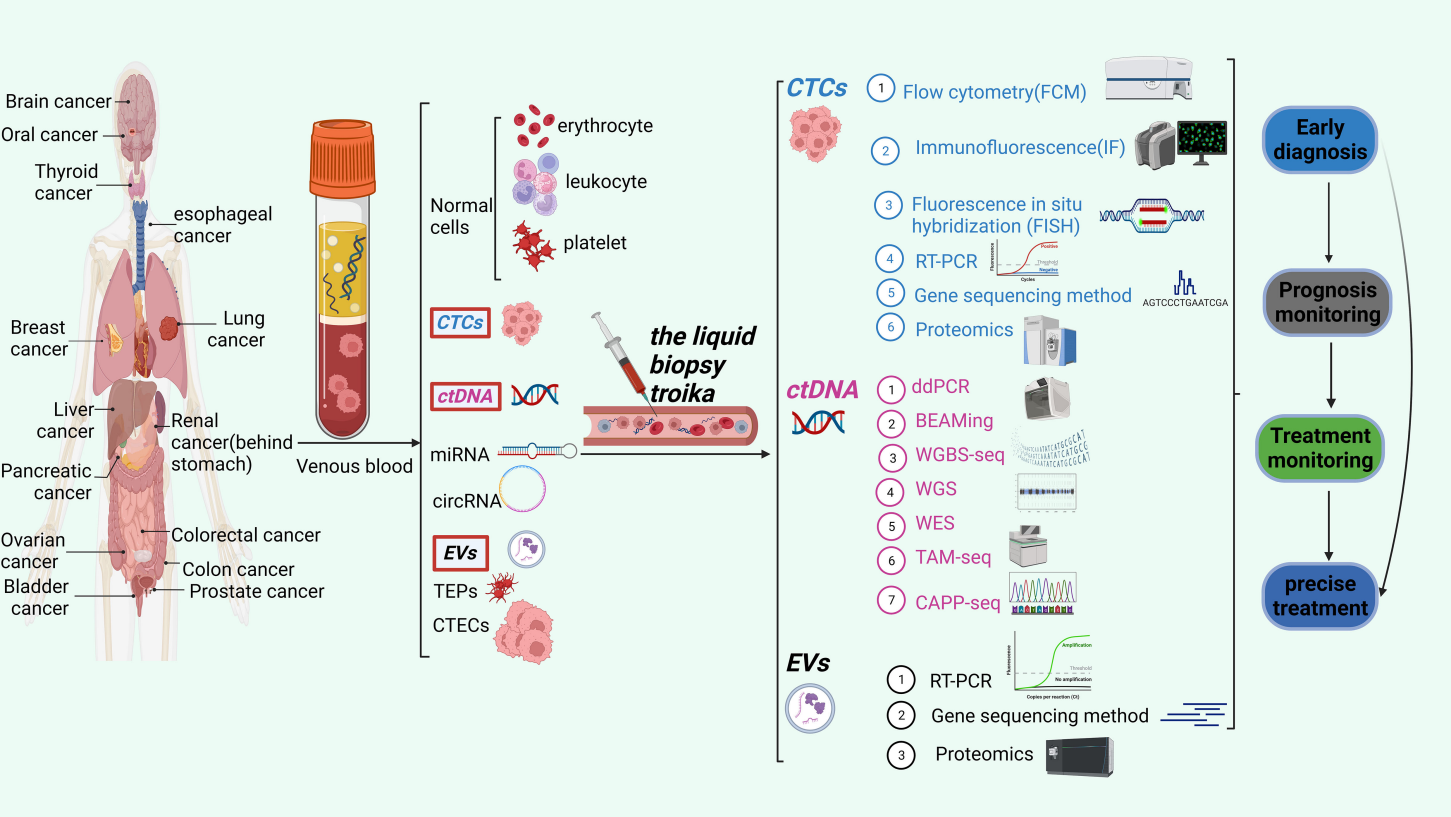
The liquid biopsy troika include CTCs, ctDNA and EVs. This figure shows the three detection techniques and their clinical applications.

In this comprehensive analysis, we thoroughly examine the latest developments in the rapidly evolving domain of liquid biopsy. Our main objective is to explore the potential uses of liquid biopsy techniques in cancer clinical diagnosis, therapy monitoring, and prognosis prediction. These techniques encompass circulating tumor cells (CTCs), circulating tumor DNA (ctDNA), and extracellular vesicles (EVs). We also discussed the benefits and drawbacks of the most recent CTC, ctDNA, and EV detection, capture, and downstream analysis technologies. The application of comprehensive liquid biopsy combined with CTC, ctDNA, and EVs detection in cancer monitoring. Finally, we discussed liquid biopsy’s potential applications and difficulties in precision medicine. **Figure 1** shows CTCs、ctDNA, and EVs detection techniques and their clinical applications.

## 2 Technologies for CTCs、ctDNA, and EV detection

### 2.1 CTCs

CTCs are tumor cells that, spontaneously or potentially resulting from surgical procedures, invade the peripheral blood circulation system after separating from the metastatic site or primary tumor. Circulating tumor cells (CTCs) have been detected in various malignancies, including breast, lung, ovarian, prostate, lung, and colon cancers. As a nearly non-invasive detection method, CTC can dynamically monitor the progression and changes of tumor conditions, has significant value for tumor diagnosis, treatment, and monitoring (20), and promises to advance precision medicine and help fundamental cancer research (21).

Cells are a more comprehensive biological entity and can provide dynamic information on RNA, proteins, DNA, and other biological molecules compared to ctDNA and EVs. This gives them an unmatched edge in assessments of transcriptomics, proteomics, and signal colocalization (22). Additionally, they can be cultivated *in vitro* to produce CTC lines for further study into therapy, the mechanisms underlying metastatic disease, and prevention (23). It has been found that CTC has differences in various aspects, such as cell size and morphology, molecular phenotype, activity level, metastasis potential, and proliferation potential. CTC detection has gradually moved from earlier cell counting to complete analysis of cell counting mixed with molecular typing due to the advancement of CTC research and increased clinical demand. CTC is extremely rare, with approximately one billion blood cells possibly being CTC. This poses significant technical challenges to isolate CTCs (23) effectively. Two counting processes comprise most CTC detection processes: CTC enrichment and separation and CTC identification. The term “CTC enrichment and separation technology” describes the process of concentrating and separating CTC from a large number of blood cells in peripheral blood based on one or more distinctive properties of CTC. CTCs were separated through biomarker-driven cell capture, employing various enrichment techniques rooted in biophysical and other characteristics, including immunogenicity, positive and negative enrichment, as well as enrichment strategies relying on size, density, dielectric properties, and deformability (14) (**Table 1**). CTC identification methods mainly include cell morphology identification, immunofluorescence staining, Fluorescence *in situ* hybridization, PCR etc (**Table 2**). CellSearch^®^ is the first FDA-approved CTC separation technique that uses antibody-labeled magnetic nanoparticles to select cells that express EpCAM and fluorescence microscopy to identify keratin and DAPI positive CD45 negative cells (31). The isolation methods of CTCs are shown in **Table 1**.

**Table 1:** Comparison of Methods for Separation of CTCs.

Method		Details	Advantages	Limitations	Refs.
positive enrichment	Immunomagnetic bead separation	Using specific antibodies to specifically bind to tumor cell surface antigens (such as EpCAM, CKs)	Applicable for EpCAM or CKs-positive CTC	CTC expresses multiple surface markers and cannot obtain all features of CTC	(24)
	Microfluidics technology	CTC enrichment and separation using cell size, deformability and surface markers based on the principle of Fluid mechanics	Applicable for EpCAM or CKs-positive CTC	Unable to obtain CTC for all features	(25)
negative enrichment		Using white blood cell antigens such as CD45 and CD61 to remove white blood cell megaphagy and platelets from the blood	Easy-to-use batch separation;	Not all blood cells are positive for antigens such as CD45 and CD61, and there is a high risk of CTC loss while removing white blood cells.	(20)
Membrane filtration method		Based on cell size	Good cell integrity, not limited by cell surface markers	Low purity, small volume CTC cannot be detected	(20)
Density gradient centrifugation method		Separation of CTC by gradient centrifugation based on different densities	Can separate CK positive and negative cells with low-cost	Low separation efficiency	(24)

**Table 2:** CTCs identification technology.

Method	Details	Advantages	Limitations	Refs.
Flow cytometry (FCM)	Quantitative analysis for individual cells	Fast detection speed; Simultaneous multi-channel detection; High sensitivity as it can detect small changes on individual cells; High specificity as it can recognize and differentiate between specific cell types by labeling cells with specific antibodies or fluorescent dyes.	Low detection sensitivity; must be a single cell suspension; can destroy cell clusters	(26)
Immunofluorescence(IF)	Identifying cells through staining signals	The staining and labeling of cells using the principle of antibody specific binding to cell surface antigens has a high degree of specificity. The detection of multiple markers of CTCs can be achieved through different antibody combinations and staining methods with high sensitivity, enabling more comprehensive identification and analysis of CTCs.	Cell morphology is diverse; antigen expression is heterogeneous, and false negatives are prone to occur.	(27, 28)
Fluorescence *in situ* hybridization (FISH)	According to the principle of complementary base pairing	Molecular detection is fast, accurate and mature in clinical application; it utilizes specific probes to hybridize with DNA molecules inside the cell, which has high specificity and can accurately detect the DNA sequence of CTCs; it can also detect markers and karyotypes inside CTCs, etc., which can improve the sensitivity of detection.	Long time consuming; vulnerable to interference	(20, 29)
RT-PCR	Quantitative analysis for CTC identification	Through reverse transcription and real-time fluorescence quantitative PCR amplification, it is able to detect very low abundance of CTCs with high sensitivity; secondly, the technology utilizes specific primers and probes to amplify and detect the target mRNA with high specificity.	RNA is easily degraded, easy to pollute, prone to false positives, indirect quantification of CTC	(20)
Gene sequencing method	Check gene sequence	It can detect molecular features such as gene mutations, fusions and methylation in CTCs, and is able to detect trace amounts of CTCs in complex samples, and is therefore characterized by high sensitivity and high resolution; suitable sequencing regions and primers can be selected with high specificity.	High price; Unable to observe cell morphology	(20)
Proteomics	Precise detection of protein expression levels and modification states	Multiple proteins can be detected at the same time, realizing high-throughput analysis; through mass spectrometry analysis and other techniques, small differences in expression in CTCs can be detected with high sensitivity; through the selection of appropriate protein markers and mass spectrometry analysis methods, interference with non-target cells can be avoided with high specificity.	CTCs are characterized by heterogeneity and low abundance, and the difficulty and complexity of proteomic analysis is high	(30)

After enriching and separating CTC in the blood, effective methods must be combined for analysis and identification. The detection of CTC has evolved from counting to analysis and type, single-cell sequencing, and cell function analysis due to the ongoing development of CTC enrichment, detection, and analysis methods. By directly examining the mechanisms of drug resistance, migration, and recurrence in each CTC, single-cell genomics and transcriptome analysis can aid in early cancer detection and provide better recommendations for early cancer diagnosis. It can also help to personalize treatment. These benefits include discovering the dynamic changes of tumors, analyzing tumor growth rules, and more (32). **Table 2** lists CTC identification techniques.

### 2.2 ctDNA

Apoptosis and necrosis of human cells will release fragmented DNA into body fluid, called cfDNA, which includes the DNA of tumor cells and the DNA of normal cells; the latter is called ctDNA. Cancer patients’ cell-free DNA has been found to include ctDNA, allowing for the analysis of the genetic abnormalities that characterize each patient’s cancer (22). Over the past 10 years, research has shown considerable promise as a possible clinical tool to promote personalized therapy. Most ctDNA fragments are double-stranded and have lengths between 160 and 200 base pairs, or roughly the same as a nucleosome (33). Numerous aspects of the tumor, including its location, metastasis, size, tumor status, vascular infiltration and staging, impact the amount of ctDNA that enters the bloodstream. As a result, there is a wide variation in the proportion of ctDNA (32). Free DNA released by blood cells and another somatic cell may produce an interference phenomenon that must be effectively distinguished. Therefore, distinguishing ctDNA from normal cfDNA is essential to reflect the disease status (34, 35) accurately.

ctDNA can provide tumor-specific genetic variation (Point mutation, insertion and deletion, rearrangement, fusion, etc.) and epigenetics (methylation, etc.) and copy number variation information; its application value in the whole process management of tumor patients has been recognized and has been written into several clinical diagnosis and treatment guidelines. The main obstacles facing ctDNA detection technology are increasing the sensitivity of detection technology and suppressing background noise signals because ctDNA has been isolated and analyzed using a variety of techniques, including Tagged-amplicon deep sequencing (TAm-Seq), cancer personalized profiling by deep sequencing (CAPP-Seq), whole genome bisulfite sequencing (WGBS-Seq), and droplet digital polymerase chain reaction (ddPCR) sequencing technologies.

Tumor Circulating Among the new liquid biopsy technologies, DNA analysis is the most advanced, although meaningful comparisons between studies are made difficult by the range of ctDNA assays. In addition to being beneficial for calculating copy number variants and discovering possibly rare mutations, ddPCR is only useful for screening for known mutations (36). DdPCR (32) is one of the most useful and applicable methods today for analyzing genomic mutations. BEAMing, like ddPCR, can only test for known mutations. The BEAMing screening method is reasonably sensitive and affordable (36). In addition, studies have shown inconsistent clinical diagnostic testing between BEAMing and ddPCR, but to a low extent (37). The most accurate and precise DNA methylation analysis method is WGBS-Seq, which provides resolution for a single cytosine measurement. Increasing the scale of this method for large population studies, on the other hand, is prohibitively expensive and poses major bioinformatic challenges (38). Although the approach may detect partly methylated regions in cancer cells, it has several practical limitations. The detection approach may show a reduced sensitivity during detection, for example, if DNA is present but has undergone various degrees of degradation (27, 28). Its capacity for high sequencing throughput distinguishes tag-amplicon deep sequencing (TAm-Seq), decreased sequencing duration and expenses, and the capability to concurrently sequence millions of DNA molecules. All primary mutation types, involving single nucleotide variations, deletions, rearrangements, insertions, and copy number alterations can be found using cancer-personalized profiling by deep sequencing (CAPP-Seq). It’s crucial to bear in mind that CAPP-Seq has limitations regarding the types of mutations it can discover (16, 25). Whole genome sequencing (WGS) is a comprehensive approach that examines a tumor’s complete genomic content, which can greatly aid therapeutic decision-making and reveal novel therapy plans, unusual mutations, and invisible oncogenes. However, WGS is constrained by difficulties with quality control, ethics, cost, and timing (39). To identify frequent or rare disease-related abnormalities, whole exome sequencing (WES) characterizes and analyzes all currently reported tumor mutations. However, it might have a lower sensitivity than other methods (36, 40). **Table 3** lists the benefits and drawbacks of the various detection strategies.

**Table 3:** Comparison of ctDNA detection methods.

Method	Details	Target mutation	Advantages	Limitations	Refs.
ddPCR	Absolute quantification of the initial sample	Known	High analytical specificity with a blank limit of 2 positive droplets and a minimum detection limit of 50 pg of methyl DNA per ml of plasma. hence, high sensitivity. The technique has a high specificity for the identification of EC (endometrial cancer) mainly by hypermethylation of ZSCAN12 (containing zinc finger and SCAN structural domain 12) and oxytocin (oxytocin).	Only able to detect limited genomic positions in a sample	(36, 41)
BEAMing	Bead, emulsion, amplification and magnetics	Known	It can detect one ctDNA molecule in 10,000 healthy cell DNA with very high sensitivity. Utilizes magnetic beads to adsorb free DNA and separates and screens it by flow cytometry for detection of specific mutations.	Only can detect known mutations, inexpensive	(42)
WGBS-Seq	A gold standard in DNA methylation analysis	Unknown	Detects individual cells or very low concentrations of ctDNA with very high sensitivity. Can accurately analyze the methylation status of each cytosine base with high specificity	High cost	(38)
TAm-Seq	The first sequencing method adapted to detect rare diagnosis mutations in cell-free DNA	Known and new	High sensitivity as ctDNA can be detected at low concentrations and its mutation information can be accurately determined. Meanwhile, specific amplification of the target region can be performed by specific primer design, which improves the specificity of detection.	General	(43)
CAPP-Seq	Targeted hybrid capture	Known and new	Detection and analysis of ctDNA by deep sequencing can detect low concentrations of ctDNA with high sensitivity. Through specific primer and probe design, tumor-associated variants can be accurately detected, avoiding interference with non-target cells and improving the specificity of detection.	General	(44, 45)
WGS	Deep sequencing of the entire genome	Unknown	Mutations in a single base can be detected, and even mutation frequencies below 1% can be detected with extremely high sensitivity and resolution. Through genome-wide sequencing, the sequence information of each gene can be accurately determined, including mutations, insertions, deletions, etc., with high specificity.	Low sensitivity, high cost	(46, 47)
WES	Deep sequencing of the exome	Unknown	Detects common and rare variants with high sensitivity. Focuses on sequencing exon regions with high specificity, which is more conducive to the discovery of disease-causing genes.	Low sensitivity, High cost,	(48)

### 2.3 EVs

EVs are membrane vesicles containing lipid bilayer secreted by cells into the extracellular space (30–2,000 nm), which nearly all cells actively secrete (6). Extracellular vesicles (EVs) can be divided into three classes according to the International Society for Extracellular Vesicles’ (MISEV’s) recommendations, depending on their size: microvesicles, apoptotic bodies, and tiny extracellular vesicles. The classification of these EVs is determined by their respective dimensions (49). It is reported that EVs derived from cancer cells are important in changing the tumor microenvironment and promoting tumor progression (50). They also carry distinctive molecules from parental cells, including proteins, miRNAs, mRNAs, lncRNAs, DNA, and lipids, indicating the state of the disease at the time. Surface markers mostly include CD63, CD81, CD9, TSG101, and HSP27 (51). Multiple studies have shown that EVs may be reliably found in a variety of bodily fluids, including blood (44), saliva (45), urine (46), bronchoalveolar fluid (47), breast milk (48), and semen (49). As a result, EV cargo targeting enables us to evaluate essential molecular data concerning illness status (50).

In recent years, there have been numerous methods for the detection of exosome contents, such as RT-PCR (52),genome sequencing (53),and proteomics (54). **Table 4** lists the detections of exosomal contents. **Table 5** lists the comparison of miRNA,mRNA and protein detection methods in EVs.

**Table 4:** detection methods for exosomal contents.

Methods	Details	Advantages	Limitations	Refs.
RT-qPCR	Quantification by standard curve method or internal reference method	Through the two steps of reverse transcription and PCR, the content of specific substances in exosomes can be detected with high specificity and sensitivity; if the efficiency of reverse transcription is high and the efficiency of PCR amplification is stable, then the sensitivity of RT-qPCR will be increased; if the primers are well-designed and can specifically bind to the target sequences, and if the PCR amplification conditions can effectively inhibit non-specific amplification, then the specificity of RT-qPCR will be improved.	Cumbersome experimental operation, may be affected by endogenous interfering substances, unable to detect non-coding RNA	(55, 56)
Gene Sequencing	Comprehensive detection of gene sequences, including coding and non-coding genes	With high sensitivity and specificity, it can detect small differences in exosomes; through massively parallel sequencing, molecular features such as gene mutations, fusions and methylation in exosomes can be detected, so as to identify and categorize exosomes; for the detection of exosomal contents, specific experimental design and technical parameters are usually used.	High experimental cost, complicated experimental operation, high requirements for technicians	(52, 57)
Proteomics	Comprehensively analyze proteins in exosomes, including a variety of different types and modification states of proteins	Low-abundance proteins can be detected, and relative and absolute protein quantification can be performed; through mass spectrometry and other techniques, proteins and their modifications expressed in exosomes can be detected, with high sensitivity, and small differences in expression in exosomes can be detected; through the selection of appropriate protein markers and mass spectrometry analysis methods, interference with non-target cells can be avoided, and a high degree of specificity is achieved.	Extraction and purification of exosomes is relatively difficult and susceptible to interference from other proteins and substances. Proteomics generates a large amount of data, which requires professional bioinformatics personnel to interpret and analyze.	(54, 58)

**Table 5:** Comparison of miRNA,mRNA and protein detection methods in EVs.

Contents	Methods	Details	Advantages	Limitations	Refs.
miRNA	Microarray Technology	Provides genome-wide expression profiles of miRNAs	High specificity, useful for detecting large amounts of abnormal miRNAs	Low sensitivity, not suitable for quantifying low levels of miRNAs	(59, 60)
	RT-qPCR	Ability to quantify low-level miRNAs	High sensitivity and low sample volume required	Need to find suitable housekeeping miRNA controls, which are often not easy to select	(61, 62)
	Next Generation Sequencing	Accurate genome-wide quantification of miRNAs	No primers or probes required, can detect new miRNAs	Requires specialized knowledge as data analysis and sample preparation is labor-intensive	(63, 64)
mRNA	Immunomagnetic Exosomal RNA (iMER) Technology	Combines immunomagnetic bead selection, RNA collection, and real-time fluorescence PCR	Simple, short analysis cycle, small sample requirement, high sensitivity	Sample preparation is complex, may have false positives or false negatives, and is technically challenging	(65, 66)
	Whole Genome Sequencing	Requires library construction, sequencing, and biosignature analysis steps	High resolution, comprehensive, flexible	Technically difficult, complex and costly data processing	(67, 68)
	Digital PCR	Realizes absolute quantitative analysis, capable of accurately detecting the concentration and copy number of mRNA in exosomes	High sensitivity, simple experimentation, applicable to a wide range of sample types	Complex data analysis, high staffing requirements, high cost	(69, 70)
Protein	ExoTEST ELISA	Detects and quantifies EVs purified from human plasma	Avoid complex and time-consuming exosome purification procedures	Proteins detected by Exotest are not exosome-specific, but are exclusively shared with cytoplasmic organelles, whose membranes are not recycled like plasma membrane structures	(71, 72)
	EV Array Technology	Detects EVs in a high-throughput manner	No purification required, fast, automated, cost-effective, highly sensitive, simultaneous traceability large number of proteins, small amount of sample and reagents required	Complex to analyze	(73, 74)
	Flow Cytometry	Simultaneous detection of multiple samples and low concentrations of proteins	High sensitivity, multi-parameter analysis, automation	Complex to analyze	(75, 76)
	Western Blot	Detects proteins using antibodies	Wide range of sample sources, high specificity, reproducibility	Cumbersome and time-consuming experimental process, affected by the quality of antibodies	(19, 77)
	Mass Spectrometry	Suitable for all types of proteins	Very high sensitivity, high degree of automation	High cost, high sample quality requirements, complicated experimental operation and data processing process	(6, 78)

EVs are an important research direction in liquid biopsy; the value of its potential applications has been continually tapped. Repeatable EV separation and enrichment will enable the evaluation of their biological functions. However, the key obstacles in this sector are the separation and purification of EVs, which are heterogeneous in function, size, content, and source (6). An important step in experimental research is effective extraction. Size exclusion chromatography (SEC), polymerization, the process of precipitation immunoaffinity chromatography, and microfluidics-based methods are the primary methods used to isolate and refine extracellular vesicles (EVs) (51, 52). For different purposes and objectives, different separation methods are utilized. However, polymer precipitation, size-based isolation, immunoaffinity capture and ultracentrifugation are routinely used. Every approach and strategy is employed based on EVs’ size and origins. Each has advantages and disadvantages. The advantages and disadvantages of the various separation methods are listed in **Table 6**.

**Table 6:** comparison of EVs separation methods.

Method	Details	Advantages	Limitations	Refs.
Ultracentrifugation	based on the size and density differences	not need to label EVs, avoid cross-contamination	time consumption, high cost, structural damage, aggregation into blocks, and lipoprotein co-separation	(79)
Density gradient centrifugation	purify EVs	improve the purity of EVs	reduce the sedimentation rate of EVs, resulting in a longer time	(80)
Polymer Precipitation	uses polyethylene glycol (PEG), reducing the solubility of the exosomes	easy to operate, short analysis time, processing large doses of samples	purity and recovery rate is relatively low	(81)
Size-Based Isolation Techniques (ultrafiltration and size exclusion chromatography)	ultrafiltration usually uses ultrafiltration membranes with different molecular weight cutoffs (MWCO) to selectively separate samples	low cost and high enrichment efficiency	reduce the recovery rate	(82)
	SEC is that the macromolecules cannot enter the gel pores, and the mobile phase finally elutes the small molecules	quick, easy, and low-cost	purity reduced	(83)
Immunoaffinity Chromatography (IAC)	the specific binding of antibodies and ligands	strong specificity, high sensitivity, high purity and high yield	not suitable for large-scale separation of EVs, produce interfering proteins	(81)
Microfluidistics-Based Techniques	the physical and biochemical properties of particular EV subtypes	low reagent volumes, very high purity of isolated products and short processing time	the fast and efficient production of sufficient EV quantities	(84)

## 3 Importtant areas of clinical applications of CTCs、ctDNA and EVs

CTCs, ctDNA, and extracellular vesicles (EVs) are now used in clinical settings as substitute biomarkers for various solid tumors, and numerous research have been conducted, indicating enormous potential in therapeutic applications.

### 3.1 Clinical applications of CTCs

The increased demand for CTCs as precision oncology biomarkers. CTC detection is an appealing, non-invasive method for cancer diagnosis. CTC detection has important value in early diagnosis, efficacy determination, prognosis evaluation, and treatment monitoring of tumors. CTC analysis makes real-time monitoring, further analysis, and identification of protein, RNA, and DNA molecules possible by effectiveness, non-invasiveness, and high repeatability (58, 59). Many clinical studies have evaluated the potential of CTCs to use blood samples from known cancer diagnosis patients for cancer testing (**Table 7**).

**Table 7:** CTCs, ctDNA, EVs biomarkers in various diseases.

Source	Disease	Biomarkers	Refs.
CTCs	Breast cancer	EpCAM, CK, TWIST, SNAIL1, SLUG, ZEB1, CD24/CD44,ALDH1, CD133, CEP8, CD47, PD-L1, Survivin, HER2-neu	(85–93)
	Small-cell lung cancer	EpCAM, CK, CD45, vimentin, CD45, DAPI	(94–96)
	NSCLC	EpCAM, CK, CD45, CD14	(97, 98)
	Prostate cancer	EpCAM, CK, AR-Vs, AR-V7, PSA, FGF2, vimentin	(99–103)
	Renal cell carcinoma	EpCAM, CK, Beclin vimentin, TWIST	(104, 105)
	Hepatocellular carcinoma	EpCAM, CK, vimentin, TWIST,	(106, 107)
	Pancreatic cancer	EpCAM,CK, vimentin, TWIST	(108–110)
	Gastric cancer	EpCAM, CEA, CK, CD45, CD44, FGFR2, HER2	(111–114)
	Colorectal cancer	EpCAM, CK, vimentin, TWIST, PRL-3, CK19, CEACAM5	(115–117)
	Glioblastoma	EGFR, olig 2 and CD 139	(118, 119)
	Ovarian cancer	EpCAM, Cytokeratin 7/8	(120)
ctDNA	NSCLC	EGFR, ALK, ROS1, BRAF	(121, 122)
	Breast Cancer	ESR1, PIK3CA	(123, 124)
	Prostate Cancer	GSTP1, RARB2, AR, SPOP, TP53, PTEN, RB1, APC, CDKN1B, BRCA2, PIK3R1, ATM, MYC, and SPOP	(125–127)
	Melanomas	BRAF	(128)
	Hepatocellular carcinoma	APC, ARID1A, CDKN2A, FAT1, LRP1B, MAP3K1, PREX2, TERT and TP53	(129)
	Gastric cancer	MET, FGFR2, EGFR, HER2	(130)
	Metastatic renal cell carcinoma	TP53, VHL, EGFR, NF1, ARID1A	(131)
	Ovarian cancer	TP53, BRCA1/2,	(132, 133)
	Glioblastoma	IDH1, IDH2, TP53, TERT, ATRX, H3F3A, HIST1H3B	(134)
	lymphoma	EZH2, BCL2, BCL6, and MYC	(135–137)
	Colorectal cancer	KRAS, NRAS, MET, ERBB2, FLT3, EGFR, MAP2K1, HER2	(138, 139)
EVs	Glioblastomas and pancreatic, colorectal, colon, liver, breast, ovarian, esophageal, bladder cancer and prostate cancer	miR-21	(140)
	Brain, pancreas, colorectum, colon, liver, breast, prostate cancer and esophagus cancer、lymphoma 、leukemia	miR-155, the miR-17-92 cluster, and miR-1246	(141–145)
	Liver, breast, colon, pancreatic cancer and hematologic malignancies	miR-146a and miR-34a	(141)
	breast cancer	miR-1246 and miR-21	(146)
	colorectal cancer	miR-638	(147)
	Acute myocardial infarction and heart failure	miR-499, miR-133, miR-208, miR-192, miR-194, miRNA-34a	(148–151)
	AD、PD, etc., central nervous system diseases	miR-21, miR-29, miR-219, LRP6, REST1, caveolin1	(152–155)
	Lung cancer	protein CD151	(156)
	Breast cancer	Phosphoprotein	(157)
	Pancreatic cancer	surface protein	(158)

#### 3.1.1 Early diagnosis

Studies have shown that CTCs can diagnose or detect clinically related cancers early. CTC, for example, has been identified in individuals with early stages (stage I-IIIA) cancer of the breast (60, 61); more than one form of CTC was found in 20% of stage I disease patients, 26.8% of stage II disease patients, and 26.7% of stage III disease patients (62). CTC has been detected using cell search technology in patients with non-metastatic colorectal cancer (including stage I and II) and non-metastatic prostate cancer (159, 160). According to Barriere et al. (65), 41% of T1-stage breast cancer patients and 47% of axillary lymph node-negative patients had CTC. A study conducted by Thery et al. (66) found that the incidence of positive circulating tumor cells (CTCs) was 21% in lymph node-negative breast cancer and 24% in instances of lymph node-positive breast cancer cases. Using the EpCAM-based NanoVelcro CTC chip, CTC can be detected in 60% of patients with stage II diseases, and positive CTC distinguishes pancreatic ductal adenocarcinoma patients from non-adenocarcinoma pancreatic disease patients (161). A meta-analysis comprising 18 prospective studies has demonstrated that a positive CTC (circulating tumor cell) result serves as a valuable biomarker for forecasting adverse overall survival outcomes in individuals diagnosed with early-stage non-small cell lung cancer (NSCLC) (68). In another study, 98 suspected prostate cancer patients were predicted to undergo biopsy diagnosis before biopsy, and Clinically significant cancer was highly associated with CTC detection utilizing the Parsortix isolation technique (99).

Collectively, these findings demonstrate that CTCs can be identified ahead of primary tumors in imaging investigations, highlighting the fact that the greatest obstacle facing the use of CTCs in early cancer diagnosis is, in fact, their paucity and isolation. The PROLIPSY examination, as exemplified by the study identifier NCT04556916, along with analogous examinations related to breast cancer (NCT03511859), non-small cell lung cancer (NCT02380196), colorectal cancer (CRC; NCT05127096), and pancreatic cancer (PANCAID) (70), represent a subset of the numerous clinical trials exploring the application of CTCs (circulating tumor cells) in the early detection of various cancers.

#### 3.1.2 Prognosis and treatment monitoring

CTCs have been examined extensively for their prognostic usefulness and are now recognized as an independent prognostic factor. CTCs have been found to have prognostic significance in various malignancies, including breast, prostate, colorectal, small cell, and non-small cell lung cancers (71–74), and their discovery in the bloodstream has been connected to various diseases. In addition to the only clinical CTC testing system approved by the FDA, CellSearch, there are other CTC testing systems, such as CanPatol and CTC chips (162).

The examination’s primary focus is the CTC count, with a positive threshold of ≥ 5, typically representing a poor prognosis. The persistence of CTC following therapy is associated with a poorer prognosis, and studies have demonstrated that changes in the quantity of CTC provide better predictive information than baseline CTC status (76, 77). The number of CTCs present before beginning neoadjuvant therapy hurts survival, according to a meta-analysis of 2239 breast cancer patients from 21 trials (246) (60). Furthermore, patient CTC analysis can aid in predicting low residual disease and late disease recurrence because CTCs can be seen 7-9 weeks before the disease’s clinical manifestation (163) and provide tools for early cancer detection (164, 165). A prognostic factor for a lower survival rate is an increased CTC count, or a failure to eliminate CTC during treatment (80, 81), and reduction or clearance of CTC count is associated with good treatment response (166). In addition, evaluating the abundance of CTC clusters can significantly improve the prognostic value of patients receiving treatment (167). CTC clusters indicate a bad prognosis and elevated baseline CTC levels are linked to decreased survival rates (168). Studies have shown that the molecular phenotype of CTC, including HER2 (159), CD47, and PD-L1, has significant prognostic value (85). CTC has been utilized as a helpful biomarker for assessing the effectiveness of cancer treatment in numerous clinical trials (99, 161), assisting clinical doctors in personalized treatment and drug resistance selection during tumor progression. CTCs can be used to track the progress of metastatic disease in patients with breast and colorectal cancer and prognosis, and a current NIH-sponsored clinical trial (NCT02973204) is looking into the utility of CTCs as clinical support tools in hepatocellular carcinoma (169).

Currently, many ongoing clinical trials also are studying the application of CTC in cancer prognosis and treatment monitoring, including breast cancer (NCT00382018、NCT02101385、NCT00601900、NCT01745757、NCT01701050、NCT00785291), prostate cancer (NCT01942837), pancreatic cancer (NCT01919151), colorectal cancer(NCT01442935、NCT01640405、NCT01640444) (162).

### 3.2 Clinical applications of ctDNA

With advantages such as being non-invasive, sensitive, comprehensive, and dynamic, ctDNA has shown great clinical value in tumor diagnosis and treatment. Although tissue biopsy is still the preferred method for diagnosis, non-invasive, real-time liquid biopsy has shown an increasingly important role and has been supported by guidelines in advanced lung cancer, breast cancer and other tumors. Currently, ctDNA testing has shown clinical value in initial molecular typing, prognostic staging of early tumors, predicting treatment responses, detecting MRD, guiding treatment, and discovering drug resistance mechanisms (170–173).

#### 3.2.1 Early diagnosis

ctDNA detection has also shown great promise in the early identification of cancer, opening up new avenues for developing highly targeted therapeutic therapies for cancer patients (174). Future clinical trials may further explore predictive biomarkers found using ctDNA analysis (168). The ctDNA of cancer patients can be examined for genetic changes such as mutations, loss of heterozygosity, microsatellite instability, methylation, and copy number variations (CNVs) (85, 169). These molecular hallmarks of tumors serve as useful biomarkers for cancer diagnosis, staging, and treatment. Cancers of the breast, colon, pancreas, and esophagus were all found to have increased ctDNA levels in their advanced stages compared to their preliminary stages, and this was true regardless of tumor-specific molecular characteristics (175).

##### 3.2.2 Prognosis and treatment monitoring

CtDNA collection from blood is non-invasive and repeatable over time compared to conventional tumor biopsy. It can monitor treatment response and predict real-time prognoses by assisting in early cancer diagnosis and recognizing small residual diseases or recurrence rates (176). Analyzing ctDNA from patients with metastatic colorectal cancer allows for monitoring of the disease’s temporal heterogeneity and individualized treatment, as more and more studies are showing (172, 174). Wyatt et al. found numerous genetic abnormalities, such as amplifications, mutations, and gene inactivation, in Prostate Cancer ctDNA by comparing it to matched tissue, which may be further examined in these patients. In order to prognostically and predictively stratify individuals based on their DNA, ctDNA assays may be used (175). In a prospective study of 69 patients with advanced NSCLC, those with high ctDNA levels fared significantly worse overall than those with low ctDNA levels and progression-free survival. Higher ctDNA than baseline indicates a poor prognosis (177). In various large patient cohorts, including prospective screening cohorts in high-risk groups for colorectal cancer, Luo and associates examined the methylation patterns on ctDNA. Methylation-based diagnostic scores were found and validated to distinguish colorectal cancer patients from healthy control groups in addition to prognosis ratings linked to patient survival (177).

Additionally, tumor cells from various sources may have different methylation profiles, enabling ctDNA analysis to reveal location information (129). Cell-free DNA (ctDNA) analysis has the potential to provide transcriptional information that could aid in the early diagnosis of cancers like prostate and colorectal by inferring transcription factor binding. Furthermore, ctDNA can predict tumor immune infiltration and response to systemic treatment following tumor recurrence after radical hepatectomy and help solve monitoring decisions and post-recurrence treatments (129), and tumor immune infiltration and responsiveness to systemic treatment after tumor recurrence after ctDNA can predict radical hepatectomy.

Currently, many ongoing clinical trials also are studying the application of ctDNA in cancer prognosis and treatment monitoring regarding colorectal cancer, such as TRACC (NCT04050345) and ADNCirc (NCT02813928), IMPROVE-IT2 (NCT04084249), NCI–sponsored randomized phase II/III COBRA study (NCT0406810), CIRCULATE trial (NCT04120701) and the DYNAMIC-II study (ACTRN12615000381583), the phase II/III DYNAMIC-III study (ACTRN12617001566325), and the phase II/III PROSPECT trial (NCT01515787) and in the OPRA trial (NCT02008656), CHRONOS (NCT03227926) and FIRE-4 (NCT02934529) (178). And Gastric Cancer clinical trials (NCT04947995、NCT04665687、NCT04511559、NCT05027347、NCT05029869、NCT04943406、NCT04510285、NCT03957564、NCT04817826、NCT04520295、NCT04576858) (179) and so on.

### 3.3 Clinical Applications of EVs

Exosome biology in disease is a relatively new area of research. EVs provide a rich source of biomarkers for the diagnosis and prognosis of disease. Tumor EVs are largely used in cancer because identifying cancer-predictive biomarkers in them can assist in increasing the early tumor diagnosis specificity and sensitivity. They have also made some headway in treating conditions affecting the heart, lungs, and central nervous system (53), and their application is increasingly being used to treat conditions affecting the liver (180) (180), kidney (181), and lung (181), among others. Due to their exceptional characteristics for transporting functional cargoes to diseased cells, exosomes can be therapeutic carriers at both the basic and clinical levels.

#### 3.3.1 Diagnostic potential of EVs

EVs are desirable as minimally invasive liquid biopsies because they are present in all biological fluids, are substantially concentrated in biofluids, and are secreted by all cells. According to some research, there is a small amount of DNA in EVs; this can be used to detect mutations in serum EVs associated with cancer (180, 182). EVs may include particular miRNAs or miRNA sets with diagnostic or prognostic value for cancer identification. Since EVs include miRNAs produced differently in cancer cells than normal cells, they may have a high diagnostic value and aid in early detection. Bladder and prostate cancer have been associated with increased levels of exosomal miR-21 in the urine, whereas glioblastomas, colorectal, colon, liver, breast, ovarian, and esophageal cancer have all been connected to increased levels of circulating exocrine miR-21. Tumor-suppressing miRNAs like miR-1 4 6a and miR-3 4a are associated with hematological, liver, breast, colon, and pancreatic cancer (183, 184). Colorectal cancer can be diagnosed with miR-638 (185), while exosomes generated from breast cancer cells are greatly enriched in miR-1246 and miR-21 (186). Furthermore, those experiencing acute myocardial infarction and heart failure have shown increased secretory microRNAs linked to cardiovascular ailments. MicroRNAs such as miR-499, miR-133, miR-208, miR-192, miR-194, and miR-34a (86–88, 187) belong to this group. There is great potential for the clinical identification of disorders of the central nervous system based on the unique expression patterns of miR-21, miR-29, miR-219, LRP6, REST1, and caveolin1 in exosomes (89–92). Combining different microRNAs may increase EV miR characteristics’ diagnostic and prognostic value, which are constantly associated with cancer diagnosis and prognosis (93, 94). Breast cancer patients’ circulating exosomes have been studied for their surface proteins (96), and their phosphoproteins have been suggested to have diagnostic potential (95). Also, lung cancer patients have significant EV protein CD151 expression (97).

#### 3.3.2 Therapeutic potential of EVs

Currently actively exploring EVs or as carriers for drug payloads as therapeutic agents (6). Compared to liposomes, injected exosomes can efficiently enter other cells and deliver functional cargo with minimal immune clearance when administered exogenously in mice (181, 188, 189). Tumor stroma-derived exosomes have also been associated with cancer chemotherapy resistance (183, 190), and thus targeting specific functions of exosomes can enhance response to therapy. EVs have the potential to serve as a targeted medication carrier and benefit from the advantages of natural drug delivery due to their capacity to cross biological barriers like the blood-brain barrier. Chinese herbal medicine Western medicine uses paclitaxel (PTX) (98), curcumin (100), berry anthocyanins (101), -element (102) triptolide (103), and compound Buyanghuanwu Decoction (104). Catalase (CAT) (106), doxorubicin (105), and others (53). Additionally, EVs can employ gene therapy techniques and transport gene therapy materials like DNA and RNA. For instance, oligonucleotides can mute particular genes to treat various human diseases, such as cancer or neurological disorders (107, 108). MicroRNAs (miRNAs) have been found to efficiently contribute to the degradation of target mRNA and the suppression of gene expression in receptor cells; as a result, EVs have been developed for CNS disorders and tumors to transport miRNA or small interfering RNA (siRNA) payloads. For example, preclinical tests using EVs to deliver miRNA or siRNA payloads focused on anti-cancer treatment and exploratory brain targeting in rodents with breast cancer (184), glioma (185)and pancreatic cancer (186, 187). Additionally, ligand enrichment on modified EVs can direct EVs to particular cell types or trigger or inhibit signal transduction events in receptor cells (113, 114).

## 4 Comprehensive liquid biopsy

With the advent of precision medicine, researchers are looking into liquid biopsy to monitor tumor growth in real time and direct treatment accordingly. Molecular residual disease (MRD), also called a minimum residual disease or, in some circumstances, quantifiable residual disease, refers to solid tumors that, following therapy, cannot be detected by conventional imaging or other laboratory techniques. However, detecting tumor molecular abnormalities through liquid biopsy methods such as ctDNA and CTC indicates the persistence and clinical progression of the tumor (191). According to studies, liquid biopsies can detect metastatic diseases at least 4 years before they are clinically detected, indicating that thorough liquid biopsy evaluation of CTC combined with ctDNA monitoring MRD can provide very important information for treating and treating patients with breast cancer (192). Clinical studies have also shown the combination of CTC and ctDNA to improve the sensitivity and specificity of MRD detection in hepatic carcinoma (193). A new study examined the prognostic significance of combining CTC and ctDNA analyses. The results showed that CTC and total cfDNA levels were separately and together related to PFS and OS in MBC (194). In addition, combining ctDNA analysis based on Guardant360 NGS with CTC counting in MBC can help determine the site of metastasis (195). In a recent pilot study, 16 patients with metastatic urothelial cancer were compared using matched CTC and ctDNA samples, demonstrating that CTC and ctDNA offered complementing information (196). Therefore, CTC and ctDNA can be jointly tested for clinical tumor MRD monitoring services. There are two techniques in the field of ctDNA MRD, tumor native and tumor agnostic. **Table 8** shows the advantages and disadvantages of these two techniques.

**Table 8:** Advantages and disadvantages of tumorigenic and tumor agnosticism.

source	Advantages	Disadvantages	Refs.
tumorigenic	1. ctDNA consists of DNA fragments released by tumor cells that contain tumor-specific information. 2. Unlike conventional imaging and pathology, ctDNA has higher sensitivity and specificity, making it a valuable tool for dynamic monitoring of a patient’s tumor load. In addition, ctDNA-MRD monitoring enables detection of tumor residuals or recurrence 8-12 months earlier compared to conventional methods. It allows comprehensive assessment of tumor status and early identification of tumor recurrence with a detection sensitivity of up to 0.01%. 3. ctDNA-MRD is associated with poor prognosis in patients with malignant tumors. Therefore, ctDNA is expected to be used as a biomarker for early diagnosis, treatment response detection and prognosis prediction in solid tumors. 4.Currently, there are many established techniques for detecting ctDNA MRD in blood, such as PCR-based single or multilocus detection、second-generation sequencing-based gene panel sequencin, second-generation sequencing-based whole-exome detection and multiplexed PCR assays. 5. ctDNA MRD assays, which can identify disease recurrence prior to radiographic imaging, e.g., clinically, MRD can effectively predict the risk of early postoperative recurrence in patients with CRC (colorectal cancer).	1. Blood contains high levels of tumor-associated DNA, therefore, the MRD concept is mainly applied to hematologic malignancies. 2. Because not all cancer patients can detect ctDNA in their blood, ctDNA testing has certain limitations, and ctDNA is mainly used to diagnose cancer by comparing the differences in DNA sequences, if a patient is infected with other viruses or microorganisms, then the body may also produce cfDNA, which will increase the cost of ctDNA testing and may also reduce the cost of the test. This will increase the cost of ctDNA testing to a certain extent, and may also decrease the accuracy of the test. 3. The current ctDNA test is not mature enough to be used in clinical practice. 4. ctDNA data in MRD are promising for testing in clinical applications, but most studies providing supporting evidence are small and limited in scope and need to be validated using large cohort studies. 5. No method for diagnosing early-stage cancer with ctDNA testing has been approved by the FDA. 6. The ability to release ctDNA into the plasma may vary from tumor to tumor, and therefore insufficient amounts of ctDNA may result in false negatives. 7. Under the influence of tumor heterogeneity and drug selection, the monitoring points of recurrent tumors may disappear, thus affecting the application of MRD technology in the process of tumor monitoring. 8. ctDNA MRD positivity at different stages may vary with increasing sample size. Therefore, if a limited number of samples are collected, the positivity rate will decrease.	(193, 194, 197–205)
tumor agnosticism	1. When we perform MRD testing, the number of specific variants we focus on is very small due to the limited total number of gene copies in plasma samples, which may not accurately reflect tumor characteristics. 2. Since ctDNA is usually DNA fragments released by apoptotic or necrotic tumor cells, it is unknown whether these fragments specifically reflect the status of the tumor itself, and therefore, even less so for MRD detection. 3. Whether positive ctDNA-MRD results work to improve clinical outcomes or whether ctDNA-MRD can be used to more accurately guide adjuvant therapy is unknown.	1. Certain cancers may be missed. 2. Tumor agnosticism lacks scientific basis to explain the mechanism of tumor occurrence and development. This viewpoint cannot provide effective guidance for the prevention and treatment of tumors. 3. Neglecting individual differences: tumor agnosticism ignores the individual differences of each patient and is unable to formulate personalized treatment plans according to the patient’s specific situation. This may lead to inaccuracy and insufficiency of treatment and affect the treatment effect. 4. Lack of effective treatments: Since tumor agnosticism believes that tumors are unknowable, effective treatments cannot be found. This may lead to patients missing the best time for treatment, aggravating their conditions and even endangering their lives.	(205, 206)

In recent years, it has been discovered that EVs miRNAs can act as vital information intermediaries for cancer cells and other cells, having a significant impact on the pathological development of tumors (205) and being potentially appropriate from a material aspect for different medication delivery and therapeutic uses (206). EVs can assess drug resistance, diagnose early cancers, and provide a prognosis. The key to understanding cancer may be found in the miRNAs found in EVs. A potential novel approach to treating lung cancer may involve preventing the production and release of EVs. EVs can be employed for additional study of ctDNA and miRNA discovered in The stability of nucleic acid molecules is successfully maintained by EVs, protecting ctDNA from degradation (17). ExoDX Lung (ALK), the first cancer diagnosis product ever released by EVs Diagnostics, was introduced in 2016. Real-time screening for EML4-ALK mutations in non-small cell lung cancer patients is made possible by the device’s EV detection technique, which can concurrently detect EVs RNA and ctDNA. According to the information supplied by EVs diagnostic (53), ExoDx lung (ALK), which can be utilized to assist physicians in determining if patients are amenable to targeted therapy with ALK inhibitors, has 88% sensitivity and 100% specificity in identifying non-small cell lung cancer. This is particularly true for those who cannot or do not want to perform a tissue biopsy.

Long-term high cancer mortality rates have motivated domestic and international research teams to create effective cancer therapy strategies. Liquid biopsy aims to collect biomarkers from biological tissues that are not solid in order to assess the disease status. It can sample and analyze different biogenic substances such as CTCs, nucleic acids, proteins or EVs in bodily fluids such as blood or urine (207). Currently, researchers are considering combining CTCs, ctDNA, RNA, miRNA, protein, and lipid EV cargos in diagnosing and prognosticating cancer and other diseases. A thorough understanding of the whole course of disease for each cancer patient might be provided if these technologies are combined to boost the positive and negative predictive value. **Table 9** lists the applications of CTCs, ctDNA and EVs in the diagnosis, prognosis and therapeutic monitoring of different diseases.

**Table 9:** CTCs, ctDNA and EVs in diagnosis, prognosis and treatment monitoring of different diseases.

Source	Disease	Diagnosis	Prognosis	Treatment Monitoring	Refs.
CTCs	Breast cancer	When the number of CTCs is elevated, it suggests the possible presence of breast cancer. In addition, CTCs detection can be used to assess the severity of the condition and to determine tumor stage.	High numbers of CTCs or specific types of CTCs may be associated with a poor prognosis.	A decrease in the number of CTCs or the disappearance of specific types of CTCs may indicate that treatment is effective. Conversely, if the number of CTCs increases or a new type appears, it may indicate disease progression or treatment failure.	(207, 208)
	Small-cell lung cancer	CTCs detection can provide additional information for the diagnosis of small cell lung cancer by capturing tumor cells in the circulating blood. Particularly in cases where the nature of a lung nodule is difficult to clarify or where there is a need for earlier information about changes in the disease, CTCs detection can be used as a rapid, non-invasive method to help doctors determine whether a small cell lung cancer is present.	The number and characteristics of CTCs can provide information about the prognosis of small cell lung cancer.	The effectiveness of treatment can be assessed by detecting changes in the number and characteristics of CTCs at regular intervals during the course of treatment.	(209, 210)
	Prostate cancer	CTCs detection can be used as a non-invasive diagnostic method to achieve high accuracy in the diagnosis of prostate cancer patients with PSA gray areas.	Numerous studies have confirmed CTCs count as a sensitive prognostic factor in prostate cancer patients. By detecting CTCs doctors can get a more accurate picture of a patient’s condition and prognosis and thus develop a more effective treatment plan.	By detecting changes in the number and type of CTCs, the patient’s resistance to treatment can be assessed.	(211, 212)
ctDNA	Breast cancer	The presence and characteristics of a tumor can be determined when there is a breast cancer tumor-specific gene mutation in ctDNA.	Specific gene mutations or gene expression patterns in breast cancer may indicate a patient’s prognosis.	A decrease in the amount of ctDNA or the disappearance of a specific gene mutation may indicate that the treatment is effective. Conversely, an increase in the amount of ctDNA or the appearance of new mutations may indicate disease progression or treatment failure.	(30, 213)
	Small-cell lung cancer	Testing of ctDNA can be used to assess the severity of the disease and determine tumor stage.	Certain gene mutations or gene expression patterns in small cell lung cancer may indicate a patient’s prognosis. Regular ctDNA testing can provide doctors with information about a patient’s condition and prognosis.	By detecting changes in the amount of ctDNA and gene mutations, the effectiveness of treatment can be assessed.	(214, 215)
	Prostate cancer	Deep whole genome sequencing of ctDNA can reveal features unique to each patient.	If the amount of ctDNA decreases or specific gene mutations disappear, it may indicate that treatment is effective and the patient has a better prognosis. Conversely, an increase in the amount of ctDNA or the appearance of new mutations may indicate disease progression or treatment failure and a poorer prognosis for the patient.	The effectiveness of treatment can be assessed by detecting changes in ctDNA at regular intervals during the course of treatment. If the amount of ctDNA decreases or specific gene mutations disappear, it may indicate that the treatment is effective. Conversely, if the amount of ctDNA increases or new gene mutations appear, it may indicate disease progression or treatment failure.	(216, 217)
EVs	Breast cancer	There are many biomarkers in extracellular vesicles that may include tumor-specific antigens, oncogenes and cytokines. In addition, extracellular vesicles can be used to detect the metastatic and invasive ability of tumor cells, providing more comprehensive information for diagnosis and disease assessment.	Specific breast cancer extracellular vesicle markers may indicate the patient’s prognosis, such as survival and risk of recurrence.	If the number of extracellular vesicles decreases or specific markers disappear, it may indicate that the treatment is effective. Conversely, if the number of extracellular vesicles increases or new markers appear, it may indicate disease progression or treatment failure. In addition, extracellular vesicles can be used as carriers for drug delivery, delivering active ingredients such as chemotherapeutic drugs to tumor cells, improving the stability and biological utilization of the drugs, while reducing the toxicity of the drugs to normal cells.	(218, 219)
	Small-cell lung cancer	By detecting specific proteins and RNA molecules in extracellular vesicles, such as miRNA-21 and TGF-β1 (transforming growth factor-β1), the symptoms and conditions of lung diseases can be effectively alleviated. In addition, some anti-inflammatory and repair molecules contained in EVs, such as IL-10 and HGF, can also promote the repair and regeneration of lung tissues, thus reducing the inflammatory response and fibrosis in lung diseases.	EVs can be used as drug delivery carriers to deliver active ingredients such as chemotherapeutic drugs to tumor cells, improving drug stability and biological utilization while reducing drug toxicity to normal cells. This helps to improve the prognosis of small cell lung cancer patients.	Through regular testing of EVs, disease progression or recurrence can be detected in a timely manner, so that the treatment program can be adjusted in time to improve the therapeutic effect.	(220, 221)
	Prostate cancer	By detecting the number of PSA (prostate-specific antigen)-positive EVs and PSA levels in the serum, it is possible to differentiate between patients with prostate cancer and those with benign prostatic hyperplasia. In addition, the accuracy of prostate cancer diagnosis can be improved by detecting tumor-specific markers in EVs.	There are biomarkers in EVs that respond to the prognosis of prostate cancer, such as ACTN4 (α-coactinomycin 4), PD-L1 (programmed death receptor ligand 1), Integrin αvβ3 (Integrin αvβ3), and Del-1 (Developmental endothelial locus-1), which have been correlated with the poor prognosis of prostate cancer, therefore the prognosis of patients can be assessed by detecting EVs in serum.	Knockdown of the ACTN4 gene, which is highly expressed in the exosomes of patients with CRPC (desmoplasia-resistant prostate cancer), reduces prostate cancer cell invasion and proliferation, and may improve the prognosis of limited and advanced prostate cancer as well as predict treatment resistance and tumor recurrence in patients with fatal disease.	(222, 223)

## 5 Conclusions and perspectives

Precision therapy is individualized pharmacological treatment based on specific characteristics of tumors (17). Tumor heterogeneity, variations in gene expression, and polymorphisms may all affect a patient’s response to medication differently. Precision medicine and molecular diagnosis have developed quickly due to current diagnostic technology (208). **Table 10** looks at the prospects of CTCs, ctDNA and EVs in tumor diagnostics in terms of cost, operability, simplicity and industrialization. Despite potential and challenges, liquid biopsies are at the forefront of this revolutionary approach, and the future of precision oncology is bright. It is also a relatively recent method to assist clinical decision-making for preventing, identifying, and treating human cancer.

**Table 10:** Outlook of CTCs, ctDNA and EVs in tumor diagnostics in terms of cost, operability, simplicity and industrialization.

	cost	operability	simplicity	industrialization	Refs.
CTCs	Requires the use of specific equipment and complex processing procedures, making it relatively costly. However, in terms of cost reduction, some studies are exploring this by improving existing techniques for CTCs detection, as well as developing new, more efficient methods for CTC detection. In addition, in the future, CTCs detection may be more cost-effective if it can be used more often for early tumor screening and surveillance. This is because in the early stages, the concentration of tumor markers is usually low and more sensitive assays are needed to detect them.	CTCs detection is relatively simple and easy to perform. By collecting a patient’s blood sample and utilizing specific equipment and reagents, the quantity and molecular expression profile of CTC can be quickly and accurately detected. This makes CTCs detection a suitable test for large-scale clinical applications.	Some new technological approaches, such as microfluidics, lab-on-a-chip technology and machine learning algorithms, are being applied to CTCs detection. The introduction of these technical methods makes CTCs detection more automated and intelligent, further simplifying the operation process.	From the aspect of industrialization, the prospect of CTCs detection in tumor diagnosis is positive. With the development and popularization of automated equipment, the industrialization of CTCs detection will be further accelerated. For example, some automated equipment can realize automatic processing, analysis and reporting of blood samples, which greatly improves the efficiency and accuracy of CTCs detection.	(208, 224)
ctDNA	With higher sensitivity and specificity, ctDNA testing can detect tumors earlier and thus provide better treatment options for patients. Therefore, while ctDNA testing may be more costly, it can provide better treatment outcomes for patients, thereby reducing overall treatment costs.	With high specificity and sensitivity, ctDNA testing can accurately detect the presence of tumor cells and genetic variants. Secondly, ctDNA is highly operable, and the molecular expression profile of ctDNA can be quickly and accurately detected by collecting blood samples from patients. In addition, with the continuous progress and optimization of technology, the operability of ctDNA detection is also improving. Some new technical methods are being applied to ctDNA testing to improve the accuracy and efficiency of the test.	Some new technical methods, such as high-throughput sequencing technology, mass spectrometry sequencing technology and single-molecule sequencing technology, are being applied to ctDNA testing to improve the accuracy and efficiency of testing. The introduction of these technical methods makes ctDNA testing more automated and intelligent, further simplifying the operation process.	From the industrialization aspect, the application prospect of ctDNA detection in tumor diagnosis is very broad. First, with the rapid development of bioinformatics technology, methylation detection is gradually expanding from single gene to multi-gene combinations as well as genome-wide level, which provides a technical basis for the industrialization of ctDNA detection. The analysis of whole-gene methylation profiles by machine learning and other methods can discover specific methylation molecular markers for distinguishing normal tissues from tumor tissues, which provides a guarantee for the accuracy of ctDNA detection. Secondly, ctDNA detection has the advantages of non-invasiveness, good reproducibility, high sensitivity and specificity, which makes it highly feasible for large-scale clinical application. With the continuous development and application of new technologies such as high-throughput sequencing technology, mass spectrometry sequencing technology and single-molecule sequencing technology, the industrialization of ctDNA testing will continue to improve, and it is expected to realize automated, intelligent and efficient testing processes. In addition, with the continuous development of precision medicine and the increasing demand for personalized treatment, the application prospect of ctDNA detection in tumor precision treatment will be broader. Early diagnosis of tumors, formulation of personalized treatment plans and real-time monitoring of treatment effects can be achieved through ctDNA testing, providing more accurate and personalized treatment services for tumor patients.	(199, 225)
EVs	As a tumor marker, its detection method is relatively simple and does not require complex processing procedures or expensive equipment, so the detection cost is relatively low.	Detection methods for EVs are relatively easy to perform. The number and molecular expression profile of extracellular vesicles can be detected quickly and accurately by collecting samples of body fluids such as blood and urine from patients.	In addition, the simplicity of EVs detection is increasing with the continuous progress and optimization of technology. Some new technological methods, such as microfluidics and nanofluidics, are being applied to EVs detection, and the introduction of these technological methods makes EVs detection more automated and intelligent, further simplifying the operation process.	From the perspective of industrialization, EVs have a very broad application prospect in tumor diagnosis. First of all, as the “fingerprint” of parental cells, EVs can reveal the metabolism, proliferation, migration and other information of parental cells in real time, which can provide a powerful tool for early diagnosis, typing, prognosis, concomitant diagnosis and dynamic monitoring in cancer detection. This makes EVs have very high potential value in tumor diagnosis. Secondly, with the development of EVs detection technology, a variety of biomarkers such as proteins and miRNAs originating from EVs have been discovered, which have shown good auxiliary diagnostic efficacy in tumors, neurodegenerative diseases, cardiovascular and cerebral vascular diseases, immune system diseases and many other diseases. This provides a technical basis for the industrialized application of EVs in tumor diagnosis. In addition, EVs are one of the most promising candidates in nanomedicine due to their biocompatibility, biodegradability, low toxicity and non-immunogenicity. Studies such as drug delivery and therapy using EVs have also achieved excellent results. This offers the possibility of industrialized application of EVs in tumor therapy.	(223, 226)

Although CTC analysis has several clinical limitations, it has the potential to deliver more thorough and accurate information on tumors. Individual differences and the extremely low CTC content in peripheral blood necessitate further research into effective CTC enrichment approaches to increase the reliability of CTC detection (23). Secondly, it is not possible to determine the appropriate CTC detection time. The morphological, genetic, and functional properties of CTCs differ due to various timeframes and treatment modalities, which impacts their therapeutic application (17). Therefore, the monitoring method for CTC is significantly challenged by its rarity and heterogeneity, which limits its clinical application to a certain extent. In addition, there are unanswered questions in CTC biology, unresolved concerns regarding the application of CTC biology in clinical settings (227) and so on. The advantage of ctDNA is that it is widely and uniformly distributed in body fluids, and the standardization of detection techniques is relatively easy. However, like CTCs, tumor patients’ ctDNA content is extremely low, making missed detection possible and necessitating highly sensitive detection technology (17). After DNA amplification, it is now possible to directly, qualitatively and quantitatively examine ctDNA using well-established detection techniques such as NGS and digital PCR owing to the rapid improvement of DNA sequencing technology. The therapeutic application value of ctDNA has to be further validated through many pertinent clinical trials and standardize the detection process (209). EVs have generated much attention in liquid biopsy of cancer as a possible biomarker for cancer detection. However, their clinical limits are also impacted by the number of EVs, separation tools, and purity (210). Even though researchers from both home and abroad have made some headway in exploring different methods for separation and purification, EV research and application are still constrained by cost and efficiency; as a result, developing a trustworthy EV extraction method will be advantageous for the clinical application of EVs (211).

In conclusion, a successful combination of these technologies may be useful for tumor research, notably for in-depth analysis and possible therapeutic applications. This is true despite the advantages and disadvantages of each technology. Blood is one of the windows for studying health and diseases. If the information in organs and blood is transformed into biological data and linked to the health trajectory of the human body, it will greatly benefit human health. Leroy E. Hood’s team found metastatic biomarkers of breast cancer, pancreatic cancer, lung cancer and hematopoietic system malignancies from the proteomic changes of plasma samples of health project participants, which advanced the diagnosis of metastatic cancer by more than 10 months (228). He has always been a proposer and practitioner of 4P medicine, distinguished by participative, personalized preventative, and predictive medicine. The core premise of 4P medicine is to identify early markers of disease transformation (229). Many of these biomarker techniques, including liquid biopsy as represented by CTCs (214), are now being researched、ctDNA (5) and EVs (65). Tumor formation and incidence are complex processes, and the underlying laws are not fully understood. One significant example is the early detection of cancers, which allows localized treatment choices to eradicate primary cancer and improves survival while lowering the likelihood of recurrence. Whether it is tissue biopsy or liquid biopsy represented by CTC, ctDNA, and EVs, it is a way to gain a deeper knowledge and understanding of tumors. Suppose these methods are integrated according to the concept of integrative medicine, and comprehensive detection and integrated analysis are carried out as far as possible when conditions permit the development of a more targeted treatment plan. In that case, it may help patients benefit from the survival time and quality of life.

## Author contributions

XW: Writing – original draft. LW: Data curation, Writing – original draft. HL: Writing – review & editing. YZ: Writing – review & editing. DH: Data curation, Writing – review & editing. ML: Investigation, Writing – review & editing. XX: Formal analysis, Writing – review & editing. JH: Formal analysis, Writing – review & editing. WZ: Funding acquisition, Writing – review & editing. TZ: Funding acquisition, Writing – review & editing.

## Glossary

**Table:**

ARV7	androgen-receptor splice variant 7
ALDH1	aldehyde dehydrogenase 1
ALK	anaplastic lymphoma kinase
AR	Androgen Receptor
BEAMing	bead, emulsion, amplification and magnetics
CRC	colorectal cancer
CTC	circulating tumor cells
CTM	circulating tumor microemboli
ctDNA	circulating tumor DNA
CAPP−Seq	cancer personalized profiling by deep sequencing
CEP8	chromosome 8 centromeric probe
CK	cytokeratin
CEACAM5	carcino-embryonic antigen-like cellular adhesion molecule 5
ddPCR	droplet digital polymerase chain reaction
EpCAM	Epithelial cell adhesion molecule
EVs	extracellular vesicles
EMT	epithelial-mesenchymal transition
EGFR	Epidermal growth factor receptor
FGF2	Fibroblast growth factor 2
FGFR2	Fibroblast growth factor receptor 2
HER2	human epidermal growth factor receptor-2
miRNA	microRNA
MRD	Molecular residual disease
MBC	Metastatic breast cancer
NSCLC	non-small cell lung cancer
OS	overall survival
PFS	Progression-Free Survival
PD-L1	programmed cell death ligand-1
PRL-3	phosphatase of regenerating liver-3
TAm−Seq	tagged−amplicon deep sequencing
WGS	whole genome sequencing
WES	whole exome sequencing
WGBS−Seq	whole genome bisulfite sequencing.
